# Tailored graphical lasso for data integration in gene network reconstruction

**DOI:** 10.1186/s12859-021-04413-z

**Published:** 2021-10-15

**Authors:** Camilla Lingjærde, Tonje G. Lien, Ørnulf Borgan, Helga Bergholtz, Ingrid K. Glad

**Affiliations:** 1 MRC Biostatistics Unit, University of Cambridge, Forvie Site, Robinson Way, Cambridge, CB2 0SR UK; 2 Department of Cancer Genetics, Institute for Cancer Research, Oslo University Hospital, Ullernchausseen 70, 0310 Oslo, Norway; 3 Department of Mathematics, University of Oslo, PO Box 1053 Blindern, 0316 Oslo, Norway

**Keywords:** Graphical lasso, Weighted graphical lasso, High-dimensional inference, Network models, Genomics, Multiomics, Gene networks, Protein–protein interaction networks, Cancer genomics, Integrative analysis

## Abstract

**Background:**

Identifying gene interactions is a topic of great importance in genomics, and approaches based on network models provide a powerful tool for studying these. Assuming a Gaussian graphical model, a gene association network may be estimated from multiomic data based on the non-zero entries of the inverse covariance matrix. Inferring such biological networks is challenging because of the high dimensionality of the problem, making traditional estimators unsuitable. The graphical lasso is constructed for the estimation of sparse inverse covariance matrices in such situations, using $L_1$-penalization on the matrix entries. The weighted graphical lasso is an extension in which prior biological information from other sources is integrated into the model. There are however issues with this approach, as it naïvely forces the prior information into the network estimation, even if it is misleading or does not agree with the data at hand. Further, if an associated network based on other data is used as the prior, the method often fails to utilize the information effectively.

**Results:**

We propose a novel graphical lasso approach, the *tailored graphical lasso*, that aims to handle prior information of unknown accuracy more effectively. We provide an R package implementing the method, tailoredGlasso. Applying the method to both simulated and real multiomic data sets, we find that it outperforms the unweighted and weighted graphical lasso in terms of all performance measures we consider. In fact, the graphical lasso and weighted graphical lasso can be considered special cases of the tailored graphical lasso, and a parameter determined by the data measures the usefulness of the prior information. We also find that among a larger set of methods, the tailored graphical is the most suitable for network inference from high-dimensional data with prior information of unknown accuracy. With our method, mRNA data are demonstrated to provide highly useful prior information for protein–protein interaction networks.

**Conclusions:**

The method we introduce utilizes useful prior information more effectively without involving any risk of loss of accuracy should the prior information be misleading.

## Background

In the area of statistical multiomics, network models provide an increasingly popular tool for modelling complex multiomic associations and assessing pathway activity. With such models, the interactions between genes, proteins or other multiomics data can be captured and studied, and provide valuable insight into their functional relationships. The resulting hubs (i.e. genes or proteins with a high number of interactions) may again be used to identify central genes, functionally important proteins or pathway initiators, and thus potential drug targets [1].

Networks may be constructed from data found by high-throughput gene expression profiling technologies, such as microarray or RNA-seq [2]. With the development of high-throughput multiomic technologies, large, genome-wide data sets have been made available. This enables the development of complex models integrating a variety of biological resources [3, 4]. By integrating several sources of multiomic data into a model, we can increase statistical power while providing further insight into complex biological mechanisms.

One setting where integrative network analysis has a lot of potential is when there are two types of data, e.g. measured mRNA and protein, associated with the same genes. A specific mRNA molecule is transcribed from each gene, which then can be translated into a specific protein. Thus, each gene is associated with a specific mRNA sequence and protein. With a proper model formulation, we could use information about the inferred network of one data type to improve graph inference on the other.

In this paper, we propose a novel approach to data integration in network models. The paper is organized as follows. In the remaining parts of this section, we discuss existing methodologies and the challenges we wish to address. In “Results and discussion”, we describe our proposed methodology, and demonstrate its performance with both simulated and real multiomic data sets. We highlight our main findings in “Conclusions”, and finally give the details of our data analyses in the “Methods” section.

### Gaussian graphical network models

In a gene network model, each gene is represented by a node and an edge between two nodes represents an association between the corresponding genes. Letting each gene be associated with some measurable molecular unit (e.g. the mRNA or protein it encodes), a graph may be constructed from observed values of these *node attributes*. The attributes, each corresponding to one of *p* genes, are represented by the multivariate random vector $(X_1, \ldots , X_{p})^T$, and a graph may be inferred from observed values of it by assuming an appropriate model.

By assuming that the vector of node attributes is multivariate Gaussian, with an unknown mean vector ${\varvec{\mu }}$ and an unknown covariance matrix ${\varvec{\Sigma }}$, a *partial correlation network* may be inferred by estimating the inverse covariance matrix, or *precision matrix*, ${\varvec{\Theta }}={\varvec{\Sigma }}^{-1}$. Given the entries $\theta _{ij}$ of $\varvec{\Theta }$, the *partial correlation* between nodes, or variables, *i* and *j* conditioned upon all others is given by

1 $$\begin{aligned} \rho _{ij\vert V\backslash \{i,j\} } = - \frac{\theta _{ij}}{\sqrt{\theta _{ii}\theta _{jj}}} \end{aligned}$$

where *V* is the set of all node pairs [5]. Since correlation equal to zero is equivalent to independence for Gaussian variables, a conditional independence graph may be constructed by determining the non-zero entries of the precision matrix ${\varvec{\Theta }}$ and assigning edges to the corresponding node pairs. The resulting model is a *Gaussian graphical model*, with the edges representing conditional dependence.

In the Gaussian graphical model framework, the edges are assumed to be undirected and unweighted. Under these assumptions, the likelihood of the data may be derived [6]. We let ${\varvec{X}}$ be the $n\times p$ matrix of observed data, with each row corresponding to one of *n* observations of the multivariate random vector of attributes. Letting ${\varvec{S}}$ be the empirical covariance matrix, ${\varvec{\Theta }}$ then has the following profile log-likelihood:

2 $$\begin{aligned} l_p({\varvec{\Theta }}) = -\frac{n p}{2} \log {(2\pi )} + \frac{n}{2}\log {(\det {\varvec{\Theta }})} - \frac{n}{2} \text{tr}\left( {\varvec{S}}{\varvec{\Theta }}\right) \end{aligned}$$

where tr denotes the trace and det the determinant. The maximum likelihood estimate for ${\varvec{\Theta }}$ is then the solution to the problem

3 $$\begin{aligned} \widehat{{\varvec{\Theta }}} = \mathop {{\mathrm{arg\,max}}}\limits _{{\varvec{\Theta }} \succ 0} \left \{\log (\det {\varvec{\Theta }})- \text{tr} ({\varvec{S}} {\varvec{\Theta }}) \right \} \end{aligned}$$

where ${\varvec{\Theta }} \succ 0$ is the requirement that ${\varvec{\Theta }}$ is positive definite.

In the graphical lasso, the graph is also assumed to be *sparse*. This means that it has a small edge-to-node ratio, or that the precision matrix has mostly zero elements. The sparsity of a graph can be measured by the number of edges $N_e$ relative to the number of possible edges, given by $\frac{2N_e}{(p^2 - p)}$.

We can not expect estimated precision matrix elements to be exactly equal to zero for real data. Further, in high-dimensional settings where the number of observations is much smaller than the number of elements to estimate, the empirical covariance matrix is not of full rank and so its inverse is not even possible to estimate directly. Thus, dimension reduction is necessary to achieve sparsity, and to estimate the precision matrix.

### The graphical lasso

*The graphical lasso* performs sparse precision matrix estimation by imposing an $L_1$ penalty on the matrix entries [6]. It is constructed to solve a penalized version of the log-likelihood problem (3),

4 $$\begin{aligned} \widehat{{\varvec{\Theta }}} = \mathop {{\mathrm{arg\,max}}}\limits _{{\varvec{\Theta }} \succ 0} \Big \{\log (\det {\varvec{\Theta }}) - \text{tr} ({\varvec{S}} {\varvec{\Theta }}) - \lambda \Vert {\varvec{\Theta }}\Vert _1 \Big \}, \end{aligned}$$

where $\Vert \cdot \Vert _1$ is the $L_1$ norm and $\lambda$ a penalty parameter that must be tuned [7]. Due to the $L_1$ penalty, the method sets many elements of $\widehat{\varvec{\Theta }}$ to zero. $\lambda$ controls the sparsity, and a larger value of it results in fewer included edges. Utilizing the fact that solving problem (4) is equivalent to iteratively solving and updating a lasso least-squares problem [7, 8], the graphical lasso algorithm is both exact and computationally efficient [6]. The method is implemented in the R packages glasso [9] and huge [10], with the latter providing several routines for penalty parameter selection.

### Penalty parameter selection

There are several penalty parameter selection methods available, and we have used two of the more common ones in our analyses.

#### StARS

Stability Approach to Regularization Selection (StARS) is a selection method based on model stability [11]. The method starts with a large penalty $\lambda$ corresponding to an empty graph, i.e. a graph with no edges, and decreases it stepwise. For each value of $\lambda$ many random subsamples are drawn from the data, and the graphical lasso is used to fit a graph for each sample. As a measure of the instability of each edge under the subsampling, the average number of times any two graphs disagree on the edge is found. By averaging this instability measure for all edges, the total instability is found.

Given a cut point $\beta$ for the instability we are willing to accept, the corresponding $\lambda$ is selected as the optimal penalty parameter. $\beta$ may be interpreted as the fraction of edges we are willing to accept as possibly wrong, and it is normally set to 0.05. This way, StARS aims to choose the least amount of regularization that makes graphs sparse as well as reproducible under random sampling. It should be noted that since the method constructs a graphical lasso graph for every subsample, it is computationally costly for large graphs.

#### The extended BIC

The extended BIC (eBIC) is a modified version of the Bayesian Information Criterion constructed for selection in high-dimensional graph settings [12]. For a given edge set *E*, it is given by

5 $$\begin{aligned} \hbox{BIC}_{\gamma }(E)= -2 l_p( {\varvec{{\hat{\Theta }}}}( E) ) + \vert E \vert \log {n} + 4 \vert E \vert \gamma \log {p} \end{aligned}$$

where $\vert E \vert$ is the number of edges in the edge set and $\gamma \in [0,1]$. If $\gamma = 0$ we get the ordinary BIC, while positive values give stronger penalization of large graphs. The parameter $\gamma$ can only be tuned by experience, and should be large enough to get a low false discovery rate, but small enough to get a satisfactorily high positive discovery rate [12].

Like for the ordinary BIC, the penalty corresponding to the model that minimizes the eBIC is chosen. This selection criterion is computationally efficient, and has been shown to outperform both the ordinary BIC and cross validation when the sizes of *p* and $\vert E \vert$ are comparable to *n*. However, when used for sparsity selection, eBIC can lead to severe over- or under-selection of edges [11]. The criterion is therefore most suitable for comparing graphs of similar sparsity.

In our applications, we are comparing relatively small graphs where the extra penalization due to high-dimensionality is not that necessary. We are comparing graphs of similar sparsity, which also means that the extra penalization of edges is not that important. In our simulations we are therefore simply choosing $\gamma =0$ so that we get the ordinary BIC, but for generality we propose to use the eBIC criterion in our method.

### The weighted graphical lasso

The weighted graphical lasso is an extension of the graphical lasso which allows the incorporation of additional information through a $p \times p$ weight matrix ${\varvec{W}}$ with entries in [0, 1]. The method was proposed in [13] and was further studied in [14].

Theoretically, weighted graphical lasso can be justified by the Bayesian interpretation of the graphical lasso, and ${\varvec{W}}$ can be regarded as a *prior weight matrix* representing prior information about the existence of edges [13, 14]. Using the *penalty matrix* ${\varvec{P}}={\varvec{1}}-{\varvec{W}}$, the estimated precision matrix must now satisfy

6 $$\begin{aligned} \widehat{{\varvec{\Theta }}} = \mathop {{\mathrm{arg\,max}}}\limits _{{\varvec{\Theta }} \succ 0} \Big \{\log (\det {\varvec{\Theta }}) - \text{tr} ({\varvec{S}} {\varvec{\Theta }}) - \lambda \Vert {\varvec{P}} \circ {\varvec{\Theta }}\Vert _1 \Big \}, \end{aligned}$$

where $\circ$ denotes component-wise matrix multiplication. (6) can be regarded as the problem (4) with prior information incorporated into the expression. It is clear that while an edge with prior weight equal to zero is not penalized at all, no edge is penalized by a factor larger than $\lambda$. Thus, edges with the minimal penalty are almost guaranteed to be included while edges with the maximum penalty are not necessarily guaranteed to be excluded, unless $\lambda$ is infinitely large.

Similarly to the original problem, optimization of the problem (6) may be done using the graphical lasso algorithm. The modification can easily be incorporated into the algorithm by replacing $\lambda$ by individual penalties $\lambda p_{ij}, \ j\ne i$, where $p_{ij}$ is the $ij^{\text{th}}$ element in ${\varvec{P}}$.

If the prior information is informative, the weighted graphical lasso is found to outperform the graphical lasso in simulations [13, 14]. However, there are several potential issues with the weighted graphical lasso. Firstly, it does not take the possibility of the prior information being partly or totally misleading into account. If an excess amount of prior information is incorrect, it can be harmful to the final estimates [13]. For example, edges corresponding to zero entries in ${\varvec{P}}$ are not penalized and therefore almost guaranteed to be included in the final model, even if this is not supported by the data.

Secondly, the weighted graphical lasso might not be able to differentiate enough between weights. The range and distribution of the weights, and thus the penalties, are not necessarily purposeful, often leading to limited effect of otherwise valuable prior information. In such a case it could be sensible to use a nonlinear transformation of the weights. This is especially important if the prior weights do not have a linear interpretation where having twice the weight indicates having twice the confidence in an edge, which could be the case if the prior weight matrix is found from the estimated precision matrix of another, related data set [13]. On the other hand, it could be that the prior information is partly informative in that it is only useful to include it to a limited degree. It is altogether clear that the prior weights should be given more consideration, and not just used naïvely in the weighted graphical lasso procedure as in [13].

Before we introduce our new weighted graphical lasso method, we would like to point out that the situation we consider differs from the one that is handled by conditional and partial Gaussian graphical models [15–17]. Also for the situations where these models are used, one has information in addition to the observed values of the node attributes. However, this information is treated as covariates that affect the conditional means of the observations, while the additional information considered in this paper informs us about possible conditional dependencies among them.

## Results and discussion

### The tailored graphical lasso

To deal with the shortcomings of the weighted graphical lasso, we propose a novel approach that aims to handle prior information of unknown accuracy and to utilize it more effectively. The idea is to use a nonlinear transformation $g_k(w)$ of the weights, where the behavior of the function $g_k(w)$ is controlled by a parameter *k* with parameter space $\Omega$. We may then choose the best transformation in the function space ${\mathcal{G}}=\{g_k(\cdot ) \vert k\in \Omega \}$ by considering the precision matrix estimates we obtain for various values of *k* in a suitable partition of $\Omega$. The optimal value $k_{\text{opt}}$ is chosen using some selection criterion. Ideally both the identity function $g_k(w)=w$ and the zero function $g_k(w)=0$ should be contained in ${\mathcal{G}}$ so that both the ordinary weighted graphical lasso and the ordinary graphical lasso is considered. This way, we attempt to avoid transformations that may result in worse estimates than these two standard methods.

**Fig. 1:**
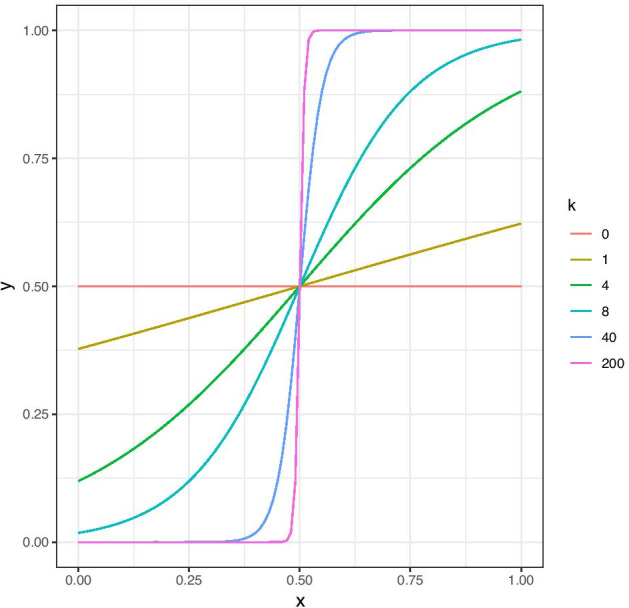
The logistic function for $w_0=0.5$ and various values of the steepness parameter *k*

Inspired by the ideas of [4], we propose a logistic function for the weight transformation. Figure 1 shows the logistic function $g_k(w) = \frac{1}{1+\exp {(-k(w-w_0))}}$, where $w_0$ is the sigmoid midpoint and *k* the steepness parameter. Evidently, for $k=0$ the function maps all weights to the same value, and we just get the ordinary unweighted graphical lasso. As *k* grows, the sigmoid function becomes more step-like. For $k=40$ it is very near being a step function and for $k=200$ it essentially is one. The transformed weights will always be mapped into [0, 1], as required for the weights in the weighted graphical lasso. The logistic function never becomes exactly the identity function $g_k(w) = w$ that would map all weights to themselves corresponding to the ordinary weighted graphical lasso. As seen from Fig. 1, it does however approximate the identity function well for $k=4$.

Thus, by appropriate data-driven tuning of the parameter *k* we may interpret a small selected *k* as an exclusion of the prior information and a large one as an inclusion and enhancement of it, giving the edges weights close to 0 or 1 depending on whether their prior weights are below or above the threshold $w_0$. This interpretability is convenient, as the optimal value of *k* found by some selection criterion then tells us how useful the prior weights are and whether increasing their differences even more improves the model.

Using the logistic weight transformation, we propose a method we call *the tailored graphical lasso*. In the algorithm, we begin by selecting the total amount of penalization. We do this with StARS, using the unweighted graphical lasso to find a common penalty parameter $\lambda$. We use StARS since it is very reliable for sparsity selection [11]. Since we only apply it once, we find that the benefits outweigh the computational loss.

For each *k*, we find the matrix ${\varvec{W}}_k$ of weights transformed with the logistic function with this steepness parameter, which gives us the penalty matrix ${\varvec{P}}_k={\varvec{1}}-{\varvec{W}}_k$. We then choose the penalty parameter $\lambda _k$ in the tailored graphical lasso that preserves the amount of penalization selected for the unweighted graph, i.e. so that $\lambda _k \Vert {\varvec{P}}_k\Vert _1 = \lambda p^2$. By preserving the amount of penalty, we achieve similar sparsity for each *k* without having to repeatedly perform StARS, which is computationally exhaustive.

As a criterion for choosing *k*, we use the eBIC as it is computationally efficient. The choice of this criterion is justified because we are comparing graphs of similar sparsity, hence severe over- or under-selection is not a concern. In the criterion, we choose a value of $\gamma \in [0,1]$ that reflects how concerned one is with false discoveries. The larger it is, the more we penalize larger graphs.

In many applications, including our simulations and applications, the prior weight matrix is found from the graphical lasso precision matrix estimate of another, related data set [13]. In such applications we let the sigmoid midpoint $w_0$ be equal to the lower $\beta$-quantile of the non-zero prior weights, where $\beta$ is the variability threshold used in the StARS tuning of the ordinary graphical lasso graph for the *prior data*. This is usually set to a default value of 0.05. This way, we avoid having to tune a second parameter. We motivate our choice of $w_0$ by the fact that $\beta$ is the upper limit set in the StARS selection for the estimated probability of an inferred edge being wrong. This means that we can expect up to a fraction $\beta$ of the inferred edges of the graph of the prior data as tuned by StARS to be incorrect. If the prior weights are obtained in another way, the $\beta$-quantile of the weights could still be selected to reflect how confident we are in their accuracy.

The algorithm is shown in Table 1. After using the method, the common penalty parameter $\lambda _{k_{\text{opt}}}$ might be adjusted slightly to achieve the exact sparsity found to be optimal for the unweighted graphical lasso graph by StARS in step 1. The maximum value $k_{\text{max}}$ must also be chosen, and as visible from Fig. 1 it is sufficient to choose it to be 80, as the logistic function basically is a step function at that point.

**Table 1:** The tailored graphical lasso algorithm

1	Select the optimal penalty $\lambda$ for the ordinary graphical lasso problem by StARS with the desired value of $\beta$ (we propose $\beta =0.05$). Let the sigmoid midpoint $w_0$ be equal to the lower $\beta$-quantile of the non-zero weights. Choose a maximum value $k_{\text{max}}$ to consider, such as 80. Choose a value of the edge penalizing parameter $\gamma$ in eBIC ($\text{BIC}_{\gamma }$) selection criterion
2	For a grid of $k \in [0,k_{\text{max}}]$:
	$\bullet$ Let ${\varvec{P}}_k = {\varvec{1}} - g_k( {\varvec{W}})$
	$\bullet$ Find $\lambda _k = \frac{\lambda p^2}{\Vert {\varvec{P}}_k \Vert _1}$
	$\bullet$ Find the estimated precision matrix $\widehat{{\varvec{\Theta }}}_k$ and the corresponding set $E_k$ of inferred edges, with the weighted graphical lasso using the penalty matrix $\lambda _k{\varvec{P}}_k$
	$\bullet$ Find $\text{BIC}_{\gamma }^{(k)} (E_k)= -2 l( {\varvec{{\hat{\Theta }}}}_k(E_k) ) + \vert E_k \vert \log {n} + 4 \vert E_k \vert \gamma \log {p}$
3	Let $k_{\text{opt}} = \mathop {{\mathrm{arg\,min}}}\limits _{k} \left\{ \text{BIC}_{\gamma }^{(k)} (E_k)\right\}$. The penalty matrix to be used is then ${\varvec{P}}_{k_{\text{opt}}}$, and the common penalty parameter is $\lambda _{k_{\text{opt}}}$

We have implemented the tailored graphical lasso in the R package tailoredGlasso (https://github.com/Camiling/tailoredGlasso).

### Simulated data

To evaluate the performance of the tailored graphical lasso, we have done comprehensive simulation studies in R [18]. The details are given in Methods. We have used our R package tailoredGlasso to perform the tailored graphical lasso, and the code for the data analysis is available on Github (https://github.com/Camiling/tailoredGlassoAnalysis).

**Fig. 2:**
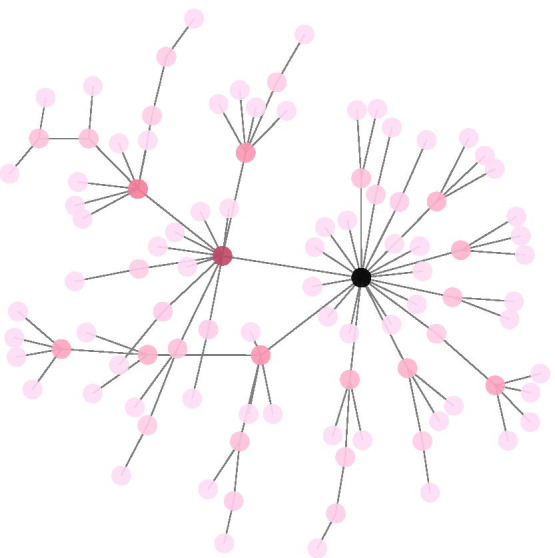
Graph structure of simulated data, with nodes colored according to their degree with darker color indicating higher degree

To make our simulations as relevant to our multiomic application of interest as possible, we have generated data with the *scale-free property*, which is a known trait in multiomic data [5]. We have simulated various sets of data with the same “true” underlying graph structure, with a sparsity of 0.02 and $p=100$ nodes. We let the sizes of the non-zero partial correlations take one of the values 0.1 or 0.2. The data sets are generated from the corresponding multivariate Gaussian distributions. Letting there be $n=80$ observations in each data set, we have a high-dimensional problem with $(p^2-p)/2=4950$ potential edges. The graph structure is shown in Fig. 2 with nodes colored according to their *degree*, the number of adjacent edges, where darker color indicates higher degree.

**Fig. 3:**
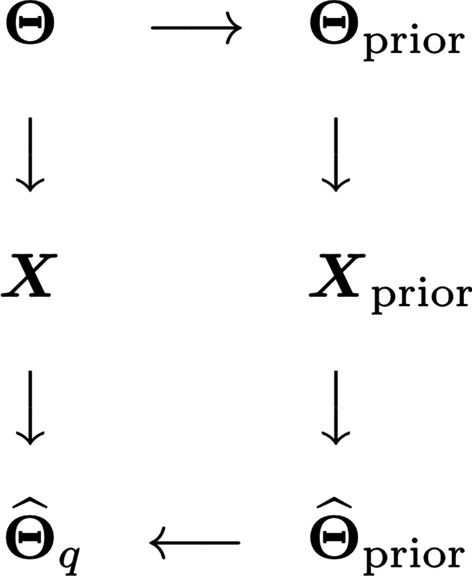
Illustration of data generation and analysis procedure in simulations. For each precision matrix ${\varvec{\Theta }}$ we consider, we modify it to create a prior precision matrix ${\varvec{\Theta }}_{\text{prior}}$. A prior data set ${\varvec{X}}_{\text{prior}}$ is generated from the resulting multivariate Gaussian distribution. The graphical lasso tuned by StARS is then used on this data to obtain a prior precision matrix estimate $\widehat{{\varvec{\Theta }}}_{\text{prior}}$, and the absolute values of the corresponding partial correlation estimates are used as weights in the tailored and weighted graphical lasso to get a precision matrix estimate $\widehat{{\varvec{\Theta }}}_q$ for the data ${\varvec{X}}$ of interest

For each data set, we have also generated various prior weight matrices in order to investigate the performance of the method in different settings. Specifically, we have created *prior precision matrices* of various similarities to the precision matrices of interest, generated *prior data* from the corresponding multivariate Gaussian distributions and used the ordinary graphical lasso tuned by StARS to estimate the prior precision matrices. Prior weight matrices are then created by taking the absolute values of the corresponding partial correlation estimates using the formula (1). The prior matrices are constructed this way to mimic real applications where we have two related data sets, both with unknown precision matrices, and we want to use information from one to improve the precision matrix estimate of the other. The precision matrix of the data used as the prior then needs to be estimated, and a prior weight matrix is constructed from it [13]. These are the prior matrices we use in the weighted graphical lasso and the tailored graphical lasso. The whole data simulation procedure is illustrated in Fig. 3.

It is important to distinguish between the accuracy of the prior precision matrix ${\varvec{\Theta }}_{\text{prior}}$ and the accuracy of the resulting prior weight matrix. As the prior partial correlations are of very small magnitude (equal to either 0.1 or 0.2), the signal is not very strong and so the inferred prior precision matrix $\widehat{{\varvec{\Theta }}}_{\text{prior}}$ (and corresponding prior weight matrix) will be much less accurate than ${\varvec{\Theta }}_{\text{prior}}$.

In our simulations, we have considered 7 different combinations of partial correlations in the network of interest and the prior, and the fraction of edges that the network of interest and the prior network disagree on. This way, we get several prior precision matrices of various accuracy and various strength of the partial correlations. For each of these cases, after the data has been generated, we have used both the unweighted and the weighted graphical lasso in addition to our proposed tailored graphical lasso to estimate the precision matrix and reconstruct the underlying graph structure. Like for the tailored graphical lasso, we selected the penalty parameter in the weighted graphical lasso so that the total amount of penalty selected for the unweighted graphical lasso graph is preserved. We have for each modified prior simulated $N=100$ corresponding data sets, and averaged the results when the above methods were applied.

The estimated graphs are assessed by the *precision*, which is the fraction of the inferred edges that are actually present in the true graph, and *recall*, which is the fraction of the edges in the true graph that are present in the inferred one. While both measures are important in understanding how well a graph reconstruction method has estimated a graph, we put more emphasis on the precision as we are more concerned about false positives than false negatives. In a multiomic setting, we would not want to identify many inaccurate interactions, but rather identify fewer but more trustworthy ones. As opposed to the precision, the recall will necessarily grow as more edges are included, and does not tell us how accurate the estimated edges that are present are.

Table 2 shows the averaged results for the different cases, where we have abbreviated the graphical lasso and weighted graphical lasso as glasso and wglasso, respectively. As expected, we see that the selected *k*, i.e. $k_{\text{opt}}$, increases with the accuracy of the prior weights.

**Table 2:** Performance of different graph reconstruction methods in simulations

Case	Edge disagreement %	Partial cor	Prior partial cor	Method	$k_{\text{opt}}$	Sparsity	Precision	Recall
1	0	0.2	0.2	Glasso	–	0.035	0.283	0.493
				Wglasso	–	0.032	0.312	0.503
				TailoredGlasso	49.64	0.031	**0**.**389**	**0**.**606**
2	0	0.2	0.1	Glasso	–	0.035	0.285	**0**.**499**
				Wglasso	–	0.034	0.293	0.493
				TailoredGlasso	13.39	0.034	**0**.**295**	0.496
3	0	0.1	0.2	Glasso	–	0.022	0.079	0.085
				Wglasso	–	0.021	0.096	0.099
				TailoredGlasso	3.34	0.021	**0**.**100**	**0**.**103**
4	0	0.1	0.1	Glasso	–	0.022	0.079	**0**.**085**
				Wglasso	–	0.020	0.082	0.083
				TailoredGlasso	5.63	0.020	**0**.**083**	0.084
5	10	0.2	0.2	Glasso	–	0.035	0.283	0.493
				Wglasso	–	0.033	0.305	0.493
				TailoredGlasso	41.2	0.032	**0**.**335**	**0**.**532**
6	20	0.2	0.2	Glasso	–	0.035	0.283	**0**.**493**
				Wglasso	–	0.034	**0**.**291**	0.491
				TailoredGlasso	4.31	0.034	**0**.**291**	**0**.**493**
7	100	0.2	0.2	Glasso	–	0.035	0.283	**0**.**493**
				Wglasso	–	0.034	0.289	0.485
				TailoredGlasso	2.94	0.034	**0**.**290**	0.485

The performance of the different graph reconstruction methods in simulations. The edge disagreement between the graph of interest and its prior, as well as the size of the partial correlations in them, is shown as well. The results are averaged over $N=100$ simulations. The best values of the different performance measures are marked in bold, and $k_{\text{opt}}$ is the mean value of the *k* chosen by the eBIC selection criterion in the tailored graphical lasso (TailoredGlasso). The graphical lasso and weighted graphical lasso are abbreviated as Glasso and Wglasso, respectively

For the most accurate prior network (case 1), *k* is selected to be as large as 49.64, and we see that the precision of the tailored graphical lasso is 37% higher than for the graphical lasso and 25% higher than for the weighted graphical lasso. In this case the recall is also 23% higher than for the graphical lasso and 20% higher than for the weighted graphical lasso. We note that the ordinary weighted graphical lasso does not perform much better than the unweighted graphical lasso. The reason is that the absolute values of partial correlations for the prior data are between 0 and 0.2, so the inclusion of these as prior weights in the ordinary weighted graphical lasso does not affect the resulting estimates too much. But by using a logistic transformation, we are able to enhance the difference of the prior weights, and this results in a much improved performance of the tailored graphical lasso

On the other hand, for completely misleading prior weights (case 7), *k* is small for the tailored graphical lasso and we get results similar to the ordinary graphical lasso. The fact that the optimal *k* is not chosen to be exactly zero can be due to randomness, where the weak inclusion of the prior information actually improves the inference.

In the other cases, the optimal *k* is larger and the tailored graphical lasso either outperforms or performs as well as the two other methods in terms of the precision.

We have also conducted a more extensive simulation study, comparing the tailored graphical lasso to five additional methods for gene network reconstruction. The methods considered are SPACE [19], ESPACE [20], neighbourhood selection (NS) [8], GeneNet [21] and CMI2NI [22]. These methods are chosen because they have been included in several similar comparative studies [20, 23]. A description of the extended simulation study, along with a discussion of the results is given in Additional file 1. A table similar to Table 2, showing the results for all methods, is given in Additional file 2.

In the extended simulation study, we find that the high dimensionality of the problem leads to problems for some of the methods, particularly the ones where the edge selection is based on false discovery rate control. With so few data points compared to the number of unknown variables, no or very few edges can be included without exceeding the FDR threshold. Thus, in the networks inferred by NS and GeneNet there are almost no edges included.

Similarly, when the signal in the data was weak with partial correlations as small as 0.1 (cases 3 and 4), the SPACE and ESPACE networks include very few edges. This means that the proposed selection criterion [12, 20] does not find the model fit of less sparse graphs to be good enough to justify the inclusion of more non-zero parameters. On the other hand, CMI2NI over-selected edges in all cases and performs worse than the tailored graphical lasso in terms of both precision and recall.

While most of the above mentioned methods tend to suffer from under-selection of edges in high-dimensional settings with weak signal in the data, the tailored graphical lasso does not have this issue. This could in part be due to its robust sparsity selection routine. Thus, in our simulated settings, where we have high-dimensional data with additional information of unknown relevance available, the tailored graphical lasso gives the overall most accurate network inference.

### Multiomic data

To illustrate possible biological applications, we have applied the tailored graphical lasso to two real multiomic data sets. Comparing the results to those from the ordinary weighted graphical lasso, we wanted to see if the tailored lasso better fits the data and can identify more multiomic interactions for which there is evidence in the literature.

We have used two data sets, the first one containing $n=743$ breast cancer tumor samples from the well-known TCGA BRCA database [24]. We considered gene expression measured by RNA-seq for $p=165$ genes, as well as their associated protein measured by Reverse Phase Protein Array (RPPA). We have only considered the genes known to encode the proteins present in the RPPA data panel. The second data set (Oslo 2) consists of $n=280$ breast cancer samples collected from hospitals in Oslo [25]. We have considered gene expression measured by microarray for $p=100$ genes, as well as their associated protein measured by RPPA.

While the research done on genomic networks using mRNA measures already is quite substantial, the field of protein–protein interaction networks is newer. It is therefore interesting to see if the large knowledge learned by gene expression analysis can be used in the construction of a protein network. Thus, we will for each of the two data sets infer protein–protein interaction networks from the RPPA data, treating the mRNA data as prior information.

The prior weights are constructed by using the graphical lasso tuned by StARS to estimate the precision matrices of the mRNA data, and letting the weights be the absolute values of the corresponding partial correlations. The partial correlation between two genes gives us the correlation between their expressions when conditioning upon all other genes. This approach results in less penalization of edges between proteins whose associated genes are found to have a strong relationship in the corresponding mRNA network. The resulting partial correlation weights are in the range [0, 0.2], just as for our simulated data. It should be noted that for genomic data, partial correlations will indeed tend to lie in this range so a partial correlation of 0.2 can be considered large (see for example [26–28]).

The distributions of the prior weights of the simulated and real multiomic data are also very similar, which also indicates that our simulations were close to the application of interest (see Additional file 3).

After the precision matrices of the RPPA data are estimated using the different methods, we assess how well they fit the data by computing the corresponding multivariate Gaussian log likelihoods (2). Since this log likelihood will depend on the sparsity of the estimated graph, and generally increases with the number of included parameters (edges), we have forced the graphs estimated by the different methods to have the same sparsity as was found optimal by StARS on the unweighted graph. This way, direct comparison is possible.

Table 3 shows the results for the TCGA data. As we see, the log likelihood of the estimated precision matrix is indeed largest for the tailored graphical lasso estimate. The results for the Oslo 2 data set are shown in Table 4, and also here the tailored graphical lasso estimate has the largest log likelihood. For both data sets, the increase in the log likelihood when comparing the tailored graphical lasso to the weighted graphical lasso is much larger than when comparing the weighted graphical lasso to the ordinary graphical lasso.

**Table 3:** Comparison of results for the TCGA data set, with the highest log-likelihood value in bold

Method	*k*_opt_	Sparsity	*l*_*p*_$(\widehat{{\varvec{\Theta }}})$
Glasso	–	0.034	− 162226.8
Wglasso	–	0.034	− 161928.4
TailoredGlasso	127.00	0.034	− **160697**.**1**

**Table 4:** Comparison of results for the Oslo 2 data set, with the highest log-likelihood value in bold

Method	*k*_opt_	Sparsity	*l*_*p*_$(\widehat{{\varvec{\Theta }}})$
Glasso	–	0.026	− 38489.65
Wglasso	–	0.026	− 38419.44
TailoredGlasso	149.0	0.026	− **38132**.**04**

The optimal *k* selected by the tailored graphical lasso is very large in both cases, meaning the method finds that the information from the mRNA networks improves the inference on the protein networks. For such large values of *k*, prior weights are mapped to values close to either 0 or 1, depending on whether they are above or below the sigmoid midpoint $w_0$. This is an interesting result, as it means that the mRNA networks are found to provide very useful information about the protein networks.

The resulting tailored graphical lasso graphs are shown in Additional files 4 and 5 for the TCGA and Oslo 2 data sets respectively. The corresponding edge lists are given in Additional files 6 and 7.

#### Biological validation and interpretation

To investigate whether the edges in the graphs found by the tailored graphical lasso have more evidence in the literature than the ones found by the ordinary weighted graphical lasso, we have also performed data mining using the STRING database, which contains known and predicted protein–protein interactions [29]. Because predicted interactions are not suitable for validation purposes, we only considered the experimentally validated interactions in STRING as evidence [29].

For both tailored and weighted graphical lasso, we calculated the fraction of edges with proof in the STRING database. To highlight the differences between the methods we only focused on the edges that the two methods disagreed on, which constituted 289 out of 456 edges in the TCGA data, and 103 edges out of 131 in the Oslo 2 data. Table 5 shows the percentage of the edges unique to the different graphs that have evidence in the STRING database. For both data sets there was far more evidence for the edges unique to the tailored graphical lasso graphs than those unique to the weighted graphical lasso graphs. Lists of the protein–protein interactions that the tailored graphical lasso was able to find, but not the weighted graphical lasso, are given in Additional files 8 and 9.

**Table 5:** Comparison of evidence for edges unique to each graph

Data set	Edge evidence %	
	Wglasso (%)	TailoredGlasso (%)
TCGA	3.8	**6**.**6**
Oslo 2	5.8	**15**.**5**

Comparison of evidence for edges unique to each graph, using experimentally determined interactions in the STRING database. The highest percentage of edges with evidence is in bold

We show here that our tailored graphical lasso method effectively identified proteins encoded by key breast cancer genes as hubs in the resulting networks from both breast cancer cohorts, such as Cellular Communication Network Factor 1 (CCN1), Estrogen Receptor 1 (ESR1) and Checkpoint Kinase 1 (CHEK1). It is also reassuring that many hubs and edges overlapped between Oslo2 and TCGA even though the two models included non-overlapping protein lists. For instance, in both cohorts, the model identified interaction between Cyclin B1 (CCNB1) and Cyclin Dependent Kinase 1 (CDK1) which are known to form Maturation-promoting factor (MPF) promoting entrance into mitosis, leading to increased proliferation of cancer cells [30, 31]. Interaction was also identified between MutS Homolog 2 (MSH2) and MutS Homolog 6 (MSH6) which dimerize to create the $\text{MutS}\alpha$ mismatch repair complex involved in DNA mismatch repair [32]. Up-regulation of mismatch repair proteins commonly occurs in cancer as a response to increased DNA damage.

In breast cancer cohorts consisting of all molecular subtypes (such as both Oslo2 and TCGA), it is expected that hubs and edges that separate basal and luminal subtypes will dominate, which the mentioned interactions are examples of, as both cell proliferation and DNA mismatch repair differ between the subtypes [33]. An even more obvious subtype specific interaction which we found in both Oslo2 and TCGA is the one between ESR1 and GATA binding protein 3 (GATA3). This interaction is typical for luminal breast tumors while not in basal since these are generally not dependent on estrogen [33].

We have shown that the tailored graphical lasso is able to identify multiple experimentally validated interactions present in the STRING database. However, using only experimentally validated interactions as evidence will inevitably produce a bias between proteins that have previously been explored in-depth and proteins that have not received as much attention. For instance, in our results we identify the transcription factor Y-Box Binding Protein 1 (YBX1) as a central hub in both Oslo2 and TCGA. This interaction is not experimentally validated in the STRING database, however YBX1 has been shown in observational studies to be relevant in breast cancer [34]. Further, it is reasonable that transcription factors are found to be hubs because of their role in controlling the rate of transcription of other genes. These findings illustrate the potential tailored lasso has for hypothesis generation.

### Extensions

In a genomic setting, prior information on pairs of genes (nodes) can be derived from a wide range of data types. In addition to the examples presented in our two biological applications, another approach is to use genome-wide association studies to gain information about so called epistatic interaction. Such interaction occurs when one mutation alters another gene’s mutation. The importance of a specific mutation pair can for instance be quantified using the number of times they are described in the literature [35], or by statistical testing [36].

As illustrated by our applications, the precision matrix of the prior data must have the same dimension as the matrix of interest, which is a limitation of the weighted graphical lasso inherited to the tailored graphical lasso. If there is a mapping between the data types, such as when we want to incorporate information from regulatory variants such as methylation or microRNA when constructing a gene expression network, this problem may however be solved by collapsing the values associated with the same gene in the prior weight matrix. This way, we get a one-to-one correspondence between the prior weight matrix and the precision matrix of interest. Collapsing the values can be done in several ways, but a simple option is to just use the mean value.

Further, the output from the tailored graphical lasso is an optimized graph which may be used as input in other methods and biological applications, for instance NegSig [37] which combines mutation data for a gene and its neighbors in a given graph.

## Conclusions

In this paper, we introduce the tailored graphical lasso as an extension to the weighted graphical lasso for graph reconstruction. The objective is to get better utilisation of the available prior information, while ensuring that the introduction of prior information may not decrease the accuracy of the resulting inferred graph. The method is implemented in the R package tailoredGlasso.

The method is developed with multiomic applications in mind, and to illustrate the performance of the method in such settings we have simulated data similar to this application. We have considered different scenarios in which the strength of the partial correlations varies both in the network of interest and the prior network, and in which the edge agreement between these networks varies. We found that if the prior information is completely useless and its inclusion in the weighted graphical lasso only results in a less accurate graph estimate, the tailored graphical lasso will give results similar to the ordinary graphical lasso and weighted graphical lasso.

On the other hand, if the prior information is informative, the tailored graphical lasso will outperform the graphical lasso and perform either as well as or better than the weighted graphical lasso. For less useful prior information the two methods will perform very similarly, and as the usefulness of the prior information increases the tailored graphical lasso will have better results as it utilizes the prior information more effectively.

Additionally, through a more extensive simulation study we find that among a larger set of methods, the tailored graphical is the most suitable for network inference from high-dimensional data with additional information of unknown accuracy available.

The method also has a nice interpretability through the estimated value of *k*, giving us a “usefulness score” for the prior information, where *k* close to zero indicates that the prior information does not provide any useful information while larger *k* indicates that it does. $k>0$ means that inclusion of the prior information to some degree is found to be useful, and $k>4$ means that enhancing the differences in the prior weights is found to be beneficial. Both for the TCGA and the Oslo 2 data set, *k* was found to be very large (127 and 149, respectively), meaning that the mRNA data was indeed found to be an informative prior for the less studied protein data.

We have applied the method to multiomic data from two studies on breast cancer, TCGA BRCA and Oslo 2, showing that mRNA data can be useful as prior information for protein–protein interaction networks. The estimated precision matrices found by the tailored graphical lasso had higher log likelihoods than the ones found by the ordinary weighted and unweighted graphical lasso. Further, through data mining with the STRING database we found that there is indeed more evidence for the graph structures found by the tailored graphical lasso than the two other methods, in particular evidence in the form of co-occurrence in literature and co-expression in large-scale analyses.

Altogether, we have seen that the tailored graphical lasso performs either as well as the ordinary weighted and unweighted graphical lasso, or better, depending on the usefulness of the prior information. This means that there is much to gain yet little to lose from using the tailored graphical lasso, as it allows us to use priors of unknown accuracy without taking risks.

## Methods

### Data simulations

#### Generating Gaussian graphs and data

In our multiomic application, the weighted graphical lasso is performed by first using the graphical lasso tuned by StARS on the mRNA data sets and RPPA data sets separately, and then using the estimated mRNA network as a prior network for the RPPA one. This is done by letting the prior weights be the absolute values of the resulting estimated partial correlations of the mRNA networks. Those estimates are found from the estimated precision matrix of the mRNA data, using formula (1). In our simulations we aim to mimic this setting, and so we will for each simulated set of data create a similar, but not identical, set of prior data.

We have done our simulations in R using the package huge. In particular, we have used the function huge.generator(), which allows us to generate data with the *scale-free property* as is a known trait in multiomic data [5]. We let all networks consist of $p=100$ vertices, and let there be $n=80$ observations of the corresponding multivariate data. The final graph has *p* edges and thus a *sparsity* of approximately 0.02. As for the values of the non-zero partial correlations, we let them all be equal to either 0.1 or 0.2.

Once a precision matrix $\varvec{\Theta }$ is constructed, huge generates the multivariate Gaussian data of the vertices from the distribution with covariance matrix $\varvec{\Sigma } = \varvec{\Theta }^{-1}$ and expectation vector ${\mathbf {0}}$.

#### Generating prior data

For a set of simulated data, we have modified its precision matrix and generated data from the resulting distribution. This way, we get sets of prior data from distributions of various similarity.

Specifically, for an initial precision matrix we have created, we have permuted a certain fraction of the edges by randomly redirecting all the edges of some nodes to others. We then consider each of those permuted precision matrices with all partial correlations being either 0.1 or 0.2, resulting in precision matrices of various accuracy and various strength of the partial correlations.

For each prior precision matrix ${\varvec{\Theta }}_{\text{prior}}$, a prior data set ${\varvec{X}}_{\text{prior}}$ is generated from the resulting multivariate Gaussian distribution. The graphical lasso tuned by StARS is then used on this data to obtain a prior precision matrix estimate $\widehat{{\varvec{\Theta }}}_{\text{prior}}$, which is used to construct a prior weight matrix by finding the absolute values of the corresponding partial correlation estimates.

#### Data analysis

For each combination of precision matrix and prior data, a precision matrix estimate $\widehat{{\varvec{\Theta }}}_q$ for the data ${\varvec{X}}$ of interest is found for each of the weight-based procedures of the tailored graphical lasso and the weighted graphical lasso. An ordinary unweighted graphical lasso estimate $\widehat{{\varvec{\Theta }}}$ of the data is also found for comparison. The whole data simulation procedure is shown in Fig. 3.

The ordinary graphical lasso was performed using the R function huge, while the weighted graphical lasso was performed using the glasso function in R as it allows for the use of a prior matrix in the penalty. We have tuned the graphical lasso graphs by StARS with instability threshold $\beta =0.05$, and selected the common penalty parameter in the weighted graphical lasso with the same weight preservation principle as in the tailored graphical lasso. According to our proposed algorithm we choose the sigmoid midpoint $w_0$ as the lower 0.05-quantile of the non-zero prior weights in the tailored graphical lasso. For simplicity, in the tailored graphical lasso we have used the eBIC with parameter $\gamma =0$ as our selection criterion. This is justifiable as we are comparing graphs of very similar sparsity, and so the extra penalization of the number of edges in the graphs is not necessary.

The results are averaged over $N=100$ simulations for all cases. The quantities we report are the precision and recall, as well as the sparsity of the estimates and the chosen *k* in the tailored graphical lasso. The details of the additional graph reconstruction methods included in the extended simulation study are given in Additional file 1.

### Multiomic data

Below we give a description of the preprossessing of the two data sets we have used, before describing the analysis we have done.

#### TCGA BRCA

The breast cancer tumor data from the TCGA BRCA database was downloaded from the UCSC Xena Browser [38]. We have used $n=743$ breast cancer tumor samples, considering gene expression measured by RNA-seq for $p=165$ genes as well as their associated protein measured by Reverse Phase Protein Array (RPPA). The RPPA data is $\log _2(x)$ transformed and median centered, while the RNA-seq data has been normalized by upper quartile FPKM and $\log _2(x+1)$ transformed [24]. The full data set is larger than the subset we have used, but to make the data applicable to our methods a reduction was necessary. First, we have disregarded “control” samples in the data set, since our interest is in the samples linked to breast cancer. We have also disregarded phosphorylated proteins in the RPPA data set, as they are only a subgroup of the fully measured proteins.

Some proteins have been measured by antibodies taken from different animals, meaning some protein measures in the data set are actually for the same protein. They are complimentary in their missingness in the sense that samples only have a measurement for one of them, and to get the complete protein profiles we therefore need to merge these values. This is done by taking their mean values after missing ones are removed. Further, there are 137 samples with many missing RPPA measurements that we discard. There are also 4 proteins whose values are missing in almost all samples, and these have been removed from the data set as well.

The full RNA-seq data set includes gene expression measurements for 60,483 genes, but we have only considered the genes known to encode the proteins present in the RPPA data set. Some samples have been split into several vials, and for those we have chosen to use vial A. Finally, the gene XBP1 was found to have all RNA-seq values equal to zero, and so we have removed this gene and its corresponding protein from the data set. This is necessary as the graphical lasso does not allow zero-variance variables.

To get a complete one-to-one correspondence between the two data types we chose to only look at the samples that were present in both data sets. Before the final analysis, the variables in the data sets were scaled and centered across samples to ensure scale-invariant penalization in the graphical lasso-based methods.

Finally, the mapping between the proteins and the associated genes that encode them was found mainly from the UCSC Xena Browser, however, not all proteins aliases were represented there. Therefore, mappings from the Stanford-Cancer Genome Atlas database [39] and the GeneCards database [40] were also used.

#### Oslo 2

The Oslo 2 data we have used includes $n=280$ breast cancer samples collected from hospitals in Oslo [25]. The gene expression for $p=100$ genes has been quantified by measuring mRNA with microarray-technology, and their associated proteins have been measured by RPPA technology. The data set containing the mRNA measurements was downloaded from the GEOquery database using the Bioconductor package in R [41], while the protein measurements are downloaded from the eprint version of the paper “Integrated analysis reveals microRNA networks coordinately expressed with key proteins in breast cancer” [25]. The microarrays are by default $\log _2(x)$ transformed, quantile normalized and hospital adjusted by subtracting from each microarray probe value the mean probe value among samples from the same hospital. This transformation and normalization is commonly done on multiomic data to get approximately normal data, as is required for the graphical lasso to work properly.

Like for the TCGA BRCA data set, some modifications of the Oslo 2 data set were necessary. First, we have only included genes in the mRNA data set that encode proteins present in the RPPA data set. To avoid violation of the independence assumption, in the case of a patient having several tumor samples from different pathological areas we have only considered the first one. Further, the measurements for two of the antibodies in the RPPA data were removed as they are known to bind to several proteins.

We have also merged some mRNA measures in the cases where there are several probes known to bind to the same gene. These are highly correlated, and so merging them is plausible. This is done by taking the mean of the scaled and centered measurements. The scaling ensures that the result is independent of the scale of the different antibodies.

Finally, the final data sets are scaled and centered, so that each gene or protein expression measurement has mean 0 and standard deviation 1 across samples.

#### Analysis

For both the TCGA BRCA and the Oslo 2 data set, we have inferred protein–protein interaction networks using both the weighted graphical lasso and the tailored graphical lasso. For each data set, the mRNA data is treated as prior information, letting the prior weights be the absolute values of its estimated partial correlations. The estimated partial correlations for the mRNA data are found from the graphical lasso estimate for the precision matrix using the formula (1).

The graphical lasso on the mRNA data was performed using the huge package in R, using StARS with instability threshold $\beta =0.05$ to select the penalty parameter. According to our tailored graphical lasso algorithm, we then choose the sigmoid midpoint $w_0$ as the lower 0.05-quantile of the non-zero prior weights in the tailored graphical lasso. We have used the eBIC with parameter $\gamma =0.6$ as our selection criterion in the tailored graphical lasso. We chose this larger value to reflect that we are more concerned about false positives than false negatives, as we will interpret the genomic implications of the results. However, as previously discussed we are comparing graphs of very similar sparsity, and so the choice of extra penalization of the number of edges in the graphs will not make a big impact.

As for the parameter *k*, we find it sufficient to consider a grid of values in [0, 150] as *k* necessarily is non negative, and since the logistic function essentially has the same step-function shape for all *k* larger than 100 as shown in Fig. 1. The weighted graphical lasso was performed using the glasso function in R, as it allows for the use of a prior matrix in the penalty.

After the precision matrices of the protein data of each data set is estimated using the different methods, we assess how well they fit the data by computing the corresponding multivariate Gaussian log likelihoods (2). Since this log likelihood will depend on the sparsity of the estimated graph, and generally increases with the number of included parameters (edges), we have forced the graphs estimated by the different methods to the sparsity found optimal by StARS on the unweighted graph. This way, direct comparison is possible.

#### The STRING database

While the fact that the tailored graphical lasso estimates have a higher log likelihood value than the weighted graphical lasso ones implies that the resulting graph explains the data better, it might be interesting to investigate the results even further. One possible way to check whether the new edges are more plausible is to check whether there are other sources supporting the existence of the gene relationships the edges represent. For this purpose, we have used the STRING database, which contains known and predicted protein–protein interactions [29]. As described on the STRING website, the interactions include direct (physical) and indirect (functional) associations and are derived from five main sources, namely genomic context predictions, high-throughput lab experiments, co-expression, automated text-mining and previous knowledge from other databases.

Because predicted interactions are not suitable for validation purposes, we only considered the experimentally validated interactions in STRING as evidence. The STRING database gives each interaction it identifies a score for each type of evidence it considers. The scores lie in [0, 1] and are indicators of confidence, i.e. how likely STRING judges an interaction to be true, given the available evidence [42]. In STRING, a score $\ge 0.4$ is considered “medium confidence”, and this threshold is proposed for determining relevant interactions [29]. We therefore only consider interactions with edge score $\ge 0.4$ in STRING as evidence.

The way we check the database for evidence of the existence of a set of edges is to feed a list of all genes involved to the STRING search engine. STRING then provides a graph where an edge between two nodes means that the database has evidence for the existence of an interaction between the two genes the nodes represent. A list of the edges in the resulting graph may then be downloaded as a .tsv file, and we can check how many of the edges in our original edge set that are present in this list. Focusing on the edges the tailored graphical lasso and the weighted graphical lasso disagree on, we may then find the fraction of edges present only in the tailored graphical lasso graph that have proof in the STRING database, and compare it to the fraction for the ones present only in the weighted graphical lasso graph. If the latter fraction is lower, the edges found by the tailored graphical lasso method have more support in the database.

## Supplementary Information

**Additional file 1**. Details on extended simulation study. A description of the extended simulation study, where more methods are included, as well as a discussion of the results.

**Additional file 2**. Table of performance in extended simulation study. The performance of the different graph reconstruction methods in the extended simulation study. The edge disagreement between the graph of interest and its prior, as well as the size of the partial correlations in them, is shown as well. The results are averaged over $N=100$ simulations. The best values of the different performance measures are marked in bold.

**Additional file 3**. Comparison of histograms of the non-zero prior partial correlation weights for the simulated data and the real multiomic data. The histograms show how the distribution of the prior weights in our simulations resemble the distribution of the prior weights used in the multiomic applications. The histograms show the non-zero prior weights for the simulated data with partial correlations equal to (a) 0.02 and (b) 0.01, and the real genomic data from (c) the TCGA data set and (d) the Oslo 2 data set.

**Additional file 4**. The tailored graphical lasso graph for the TCGA BRCA RPPA data. The graph found in the analysis of the TCGA BRCA data we did in this paper, using the RNA-seq data as prior information.

**Additional file 5**. The tailored graphical lasso graph for the Oslo 2 RPPA data. The graph found in the analysis of the Oslo 2 data we did in this paper, using the mRNA data as prior information.

**Additional file 6**. List of genes with interactions in the TCGA BRCA tailored graphical lasso graph. Each row contains the name of two genes whose nodes in the tailored graphical lasso graph have an edge.

**Additional file 7**. List of genes with interactions in the Oslo 2 tailored graphical lasso graph. Each row contains the name of two genes whose nodes in the tailored graphical lasso graph have an edge.

**Additional file 8**. List of the gene interactions in the Oslo 2 tailored graphical lasso graph that the weighted graphical lasso was not able to find. Each row contains the name of two genes whose nodes have an edge in the tailored graphical lasso graph, but not in the weighted graphical lasso graph.

**Additional file 9**. List of the gene interactions in the TCGA BRCA tailored graphical lasso graph that the weighted graphical lasso was not able to find. Each row contains the name of two genes whose nodes have an edge in the tailored graphical lasso graph, but not in the weighted graphical lasso graph.

## Data Availability

The TCGA BRCA data set analysed in this study is publicly available [24] in the University of California Santa Cruz Xena Browser repository (http://xena.ucsc.edu) [38], from where we downloaded both the BRCA RNA-Seq FPKM-UQ (https://xenabrowser.net/datapages/?dataset=TCGA-BRCA.htseq_fpkm-uq.tsv&host=https%3A%2F%2Fgdc.xenahubs.net) and the RPPA (https://xenabrowser.net/datapages/?dataset=TCGA.BRCA.sampleMap%2FRPPA&host=https%3A%2F%2Ftcga.xenahubs.net) data set. The Oslo 2 mRNA expression data are available from the Gene Expression Omnibus repository with accession number GSE58212 (https://www.ncbi.nlm.nih.gov/geo) [43]. The RPPA data from the Oslo 2 data set can be downloaded from Additional file 4 of the e-print [25] (https://genomemedicine.biomedcentral.com/articles/10.1186/s13073-015-0135-5). The tailored graphical lasso has been implemented in the R package tailoredGlasso (https://github.com/Camiling/tailoredGlasso). R code for the simulations and data analyses in this paper is available at https://github.com/Camiling/tailoredGlassoAnalysis.

## Abbreviations

mRNA: Messenger ribonucleic acid
RPPA: Reverse phase protein array
eBIC: Extended Bayesian information criterion
StARS: Stability approach to regularisation criterion
