# Supplementary material for: Tailored graphical lasso for data integration in gene network reconstruction
Source: BMC Bioinformatics. 2021 Oct 15;22:498. doi: 10.1186/s12859-021-04413-z (PMC8518261; doi:10.1186/s12859-021-04413-z)
Supplement: Supplementary file 1 — Additional file 1. Details on extended simulation study. A description of the extended simulation study, where more methods are included, as well as a discussion of the results. [file 12859_2021_4413_MOESM1_ESM.pdf]

Details on Extended Simulation Study

1 Methods

1.1 Extended simulation study

In this Additional file we give the details of the extended simulation study, which extends the one in the paper by including five other methods for network reconstruction. The data generation procedure is identical to the one used in the simulation study in the paper, and the only difference is that we now perform network reconstruction with five additional methods.

1.2 Methods included

In the extended simulation study, the five other methods we include are SPACE [1], ESPACE [2], neighbourhood selection (NS) [3], GeneNet [4] and CMI2NI [5], as these have been included in several similar comparative studies [2, 6]. For a summary of the different methods, we refer to [2].

1.3 Availability of Methods

The R package `space` implements the SPACE approach [1], while ESPACE is implemented in the R package `espace` (available at https://sites.google.com/site/dhyeonyu/software). We perform the neighbourhood selection approach using the R package `huge` [7]. The selection procedure in GeneNet is provided in the R package `GeneNet` [4]. MATLAB code for CMI2NI is available at http://www.comp-sysbio.org/cmi2ni, and to include the method in our simulation study we have translated the code to R. The translated R code is available at https://github.com/Camiling/tailoredGlassoAnalysis, along with code for the whole simulation study.

2 Parameter choices

2.1 SPACE and ESPACE

All the methods we consider require the selection of a sparsity-controlling parameter or threshold. Some of them have a default or suggested value, in which case this is the value we use. For the other methods, namely SPACE and ESPACE, there is no suggested value of the sparsity controlling parameter λ and it must be selected by

some criterion. Additionally, the parameter $0 < \alpha \leq 1$ controlling the influence of prior hub information must be selected for ESPACE.

A GIC-type criterion [8] is suggested in [2]. However, in our high-dimensional setting the criterion is always minimized by the empty graph. Therefore, we instead use the eBIC criterion [9] as this is specifically developed for high-dimensional settings. Although this is not as stable of a selection criterion as StARS [10], which is implemented in the ordinary graphical lasso, the eBIC is applicable to both SPACE and ESPACE and performs better than the proposed GIC-type criterion. To avoid the problem of under-selection in the two methods we use the minimal value $\gamma = 0$ for the additional penalty parameter in the eBIC criterion.

As suggested in [11], we consider a grid of λ values in $(20, \lambda^{\max})$ where $\lambda^{\max}$ is the smallest value of lambda that yields an empty graph. However, in the cases where the partial correlations are smaller than 0.1 we need more shrinkage in order to get positive definite precision matrix estimates. Therefore we in these simulation only consider values of λ in $(30, \lambda^{\max})$. In ESPACE the parameter α must also be selected. The eBIC score is therefore computed for a grid of λ and α values in order to identify the minimizing values (λ^*, α^*). Although it is time consuming, we perform this grid search since it is suggested by [2].

In ESPACE, prior information about hub nodes must be incorporated through a list of hub genes. To identify hubs from the prior network we use the definition in [2], where a hub is defined as a node whose degree is both greater than 7 and above the 0.95 quantile of the degree distribution.

2.2 NS, GeneNet and CMI2NI

For NS, we select the sparsity controlling penalty parameter with the approach proposed in [3], controlling the false discovery rate (FDR) at $\alpha = 0.2$. Similarly, we control the FDR in GeneNet with the default cutoff of $\alpha = 0.2$ [4]. For CMI2NI, we used the threshold value 0.03 to determine independence, as in [5] and [2].

3 Results

3.1 Summarising table

A table similar to Table 2 in the paper, but with the results from the additional five methods included, is given in Additional File 10. The table shows the performance of the different graph reconstruction methods for the seven cases considered. The

edge disagreement between the graph of interest and its prior, and the size of the partial correlations, are shown as well. The results are averaged over $N = 100$ simulations. The best values of the performance measures are marked in bold.

3.2 High-dimensional issues

In the simulated data, the $p = 100$ nodes yield $(p^2 - p)/2 = 4950$ potential edges. With $n = 80$ observations in each data set, network inference from the simulated data is a high-dimensional problem. This has evidently lead to problems for some of the methods, particularly the ones where the edge selection is based on FDR control. With so few data points compared to the number of unknown variables, no or very few edges can be included without exceeding the FDR threshold. Thus, in the networks inferred by NS and GeneNet there are almost no edges included. The other methods, which do not determine edges based on an FDR controlling threshold, did not have the same issue with severe under-selection in all cases.

3.3 Accuracy measures

As the networks estimated by NS and GeneNet had none or very few edges included, we cannot say much about their accuracy. As we see from the table in Additional file 10, if any edges have been included they often have high precision. This is expected from very sparse graphs with only a few edges, as only the edges with the strongest signal have been included. Correspondingly, the recall is very low.

When it comes to the other methods, we see that CMI2NI selects the most edges. It obtains a sparsity of 0.040 for the networks with the largest partial correlations (cases 1, 2, 5, 6 and 7) and a sparsity of 0.031 for the networks with the smallest partial correlations (cases 3 and 4). As a result, its precision is the lowest among all methods. Compared to the other more sparse networks, the recall is however not particularly high either. In most cases both the precision and the recall is in fact lower than for the tailored graphical lasso.

In most of the cases with the largest partial correlations in the network of interest, SPACE and ESPACE both result in higher precision but lower recall than the tailored graphical lasso. This can be expected since their inferred networks are sparser. In the cases where the partial correlations are smallest (cases 3 and 4), both SPACE and ESPACE result in severe under-selection of edges. While using eBIC for sparsity selection instead of the GIC-type criterion improves this issue, there

are still fewer edges included than there are in the true networks. As a result, these methods have higher precision but much lower recall than the tailored graphical lasso in the cases with weak signal (small partial correlations).

4 Discussion

While methods like NS and GeneNet can provide a quick way of inferring networks with a simple FDR-controlling selection procedure, the data we considered in our simulations were too high-dimensional for this approach to control the FDR.

Similarly, when the signal in the data was weak with partial correlations as small as 0.1, the SPACE and ESPACE networks with sparsity selected by information criteria such as the suggested GIC-type [8] and eBIC [9] included very few edges. This means that the criteria did not find the model fit of less sparse graphs to be good enough to justify the inclusion of more non-zero parameters.

CMI2NI, on the other hand, selected the most edges in all cases. This over-selection of edges resulted in low precision, and the recall was not particularly high either. In most cases both its recall and its precision was lower than for the tailored graphical lasso.

The tailored graphical lasso has a robust sparsity selection routine implemented, which ensures that it is able to infer a relatively accurate number of edges even in very high-dimensional cases where the signal in the data is very weak. The methods considered in this simulation study can be very useful, particularly ESPACE which similarly to the tailored graphical lasso can incorporate prior information in a data-driven way. The methods do however lack sparsity selection routines that give reasonably sized networks even in very high-dimensional settings with weak signal.

The tailored graphical lasso, on the other hand, does not suffer from under-selection even in very high-dimensional cases with weak signal. Further, as discussed in the paper it performs either as well as the ordinary weighted and unweighted graphical lasso, or better, depending on the usefulness of the prior information. We have also seen that the data-driven incorporation of prior information allows us to use priors of unknown accuracy without taking risks. Altogether, this makes the tailored graphical lasso very suitable for high-dimensional data with additional information of unknown relevance available.

References

1. Peng, J., Wang, P., Zhou, N., Zhu, J.: Partial correlation estimation by joint sparse regression models. Journal of the American Statistical Association **104**(486), 735–746 (2009)
2. Yu, D., Lim, J., Wang, X., Liang, F., Xiao, G.: Enhanced construction of gene regulatory networks using hub gene information. BMC bioinformatics **18**(1), 1–20 (2017)
3. Meinshausen, N., Bühlmann, P.: High-dimensional graphs and variable selection with the lasso. The Annals of Statistics **34**, 1436–1462 (2006)
4. Schäfer, J., Opgen-Rhein, R., Strimmer, K.: Reverse engineering genetic networks using the genenet package. J Am Stat Assoc **96**, 1151–1160 (2001)
5. Zhang, X., Zhao, J., Hao, J.-K., Zhao, X.-M., Chen, L.: Conditional mutual inclusive information enables accurate quantification of associations in gene regulatory networks. Nucleic acids research **43**(5), 31–31 (2015)
6. Zhang, M., Li, Q., Yu, D., Yao, B., Guo, W., Xie, Y., Xiao, G.: Geneck: a web server for gene network construction and visualization. BMC bioinformatics **20**(1), 1–7 (2019)
7. Jiang, H., Fei, X., Liu, H., Roeder, K., Lafferty, J., Wasserman, L., Li, X., Zhao, T.: Huge: High-Dimensional Undirected Graph Estimation. (2020). R package version 1.3.4.1. https://CRAN.R-project.org/package=huge
8. Yu, D., Son, W., Lim, J., Xiao, G.: Statistical completion of a partially identified graph with applications for the estimation of gene regulatory networks. Biostatistics **16**(4), 670–685 (2015)
9. Foygel, R., Drton, M.: Extended Bayesian information criteria for Gaussian graphical models. In: Advances in Neural Information Processing Systems, vol. 23, pp. 604–612 (2010)
10. Liu, H., Roeder, K., Wasserman, L.: Stability Approach to Regularization Selection (StARS) for high dimensional graphical models. In: Proceedings of the 23rd International Conference on Neural Information Processing Systems, vol. 2, pp. 1432–1440 (2010)
11. Yu, D., Son, W., Lim, J., Xiao, G.: Statistical completion of a partially identified graph with applications for the estimation of gene regulatory networks. Biostatistics **16**(4), 670–685 (2015)
