# Supplementary material for: Tailored graphical lasso for data integration in gene network reconstruction
Source: BMC Bioinformatics. 2021 Oct 15;22:498. doi: 10.1186/s12859-021-04413-z (PMC8518261; doi:10.1186/s12859-021-04413-z)
Supplement: Supplementary file 2 — Additional file 2. Table of performance in extended simulation study. The performance of the different graph reconstruction methods in the extended simulation study. The edge disagreement between the graph of interest and its prior, as well as the size of the partial correlations in them, is shown as well. The results are averaged over $N=100$ simulations. The best values of the different performance measures are marked in bold. [file 12859_2021_4413_MOESM2_ESM.pdf]

Case	Edge disagreement %	Partial cor	Prior partial cor	Method	k_{opt}	Sparsity	Precision	Recall
1	0	0.2	0.2	Glasso	-	0.035	0.283	0.493
				Wglasso	-	0.032	0.312	0.503
				TailoredGlasso	49.64	0.031	0.389	**0.606**
				Space	-	0.028	0.365	0.471
				Espace	-	0.018	0.452	0.390
				NS	-	0.001	**0.929**	0.052
				GeneNet	-	0.000	-	-
				CMI2NI	-	0.040	0.236	0.472
2	0	0.2	0.1	Glasso	-	0.035	0.285	**0.499**
				Wglasso	-	0.034	0.293	0.493
				TailoredGlasso	13.39	0.034	0.295	0.496
				Space	-	0.029	0.361	0.486
				Espace	-	0.025	0.398	0.455
				NS	-	0.001	**0.948**	0.053
				GeneNet	-	0.000	-	-
				CMI2NI	-	0.040	0.240	0.484
3	0	0.1	0.2	Glasso	-	0.022	0.079	0.085
				Wglasso	-	0.021	0.096	0.099
				TailoredGlasso	3.34	0.021	0.100	0.103
				Space	-	0.001	**0.585**	0.007
				Espace	-	0.002	0.409	0.019
				NS	-	0.000	-	-
				GeneNet	-	0.000	-	-
				CMI2NI	-	0.031	0.070	**0.107**
4	0	0.1	0.1	Glasso	-	0.022	0.079	0.085
				Wglasso	-	0.020	0.082	0.083
				TailoredGlasso	5.63	0.020	0.083	0.084
				Space	-	0.001	**0.585**	0.007
				Espace	-	0.001	0.218	0.010
				NS	-	0.000	-	-
				GeneNet	-	0.000	-	-
				CMI2NI	-	0.031	0.070	**0.107**

Case	Edge disagreement %	Partial cor	Prior partial cor	Method	k_{opt}	Sparsity	Precision	Recall
5	10	0.2	0.2	Glasso	-	0.035	0.283	0.493
				Wglasso	-	0.033	0.305	0.493
				TailoredGlasso	41.2	0.032	0.335	**0.532**
				Space	-	0.028	0.365	0.471
				Espace	-	0.018	0.452	0.394
				NS	-	0.001	**0.929**	0.052
				GeneNet	-	0.000	-	-
				CMI2NI	-	0.040	0.236	0.472
6	20	0.2	0.2	Glasso	-	0.035	0.283	**0.493**
				Wglasso	-	0.034	0.291	0.491
				TailoredGlasso	4.31	0.034	0.291	**0.493**
				Space	-	0.028	0.365	0.471
				Espace	-	0.026	0.382	0.456
				NS	-	0.001	**0.929**	0.052
				GeneNet	-	0.000	-	-
				CMI2NI	-	0.040	0.236	0.472
7	100	0.2	0.2	Glasso	-	0.035	0.283	**0.493**
				Wglasso	-	0.034	0.289	0.485
				TailoredGlasso	2.94	0.034	0.290	0.485
				Space	-	0.028	0.365	0.471
				Espace	-	0.025	0.384	0.456
				NS	-	0.001	**0.929**	0.052
				GeneNet	-	0.000	-	-
				CMI2NI	-	0.040	0.236	0.472
