# Supplementary figures and images for: Tailored graphical lasso for data integration in gene network reconstruction
Source: BMC Bioinformatics. 2021 Oct 15;22:498. doi: 10.1186/s12859-021-04413-z (PMC8518261; doi:10.1186/s12859-021-04413-z)

(a)

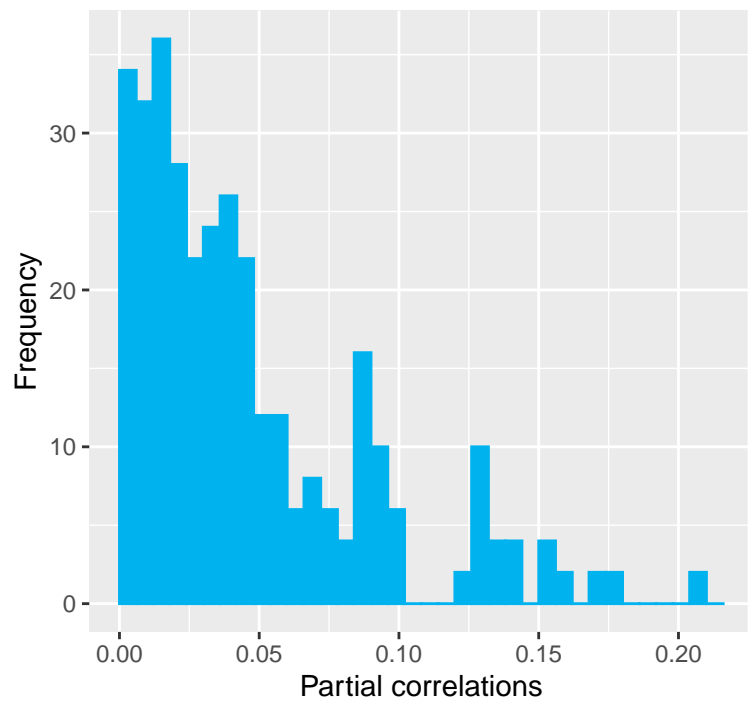

(b)

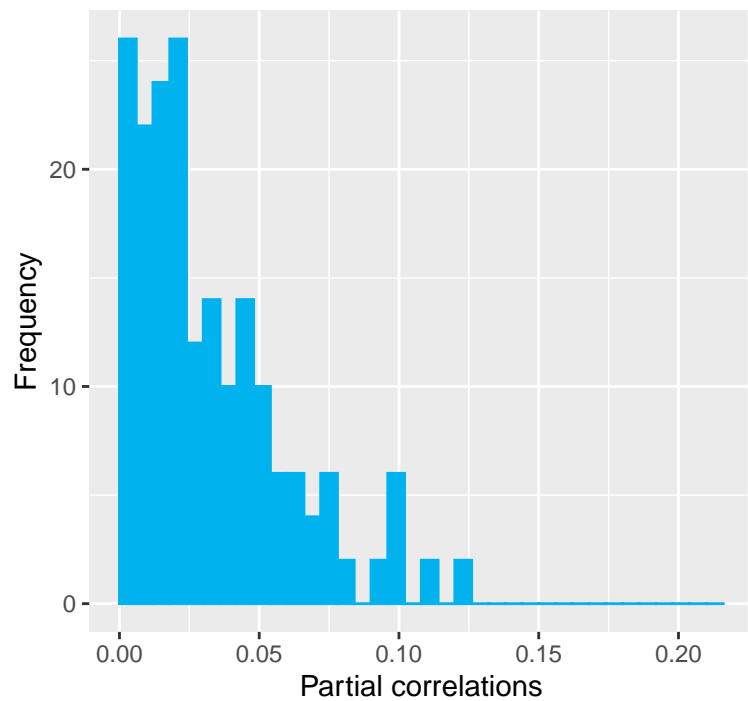

(c)

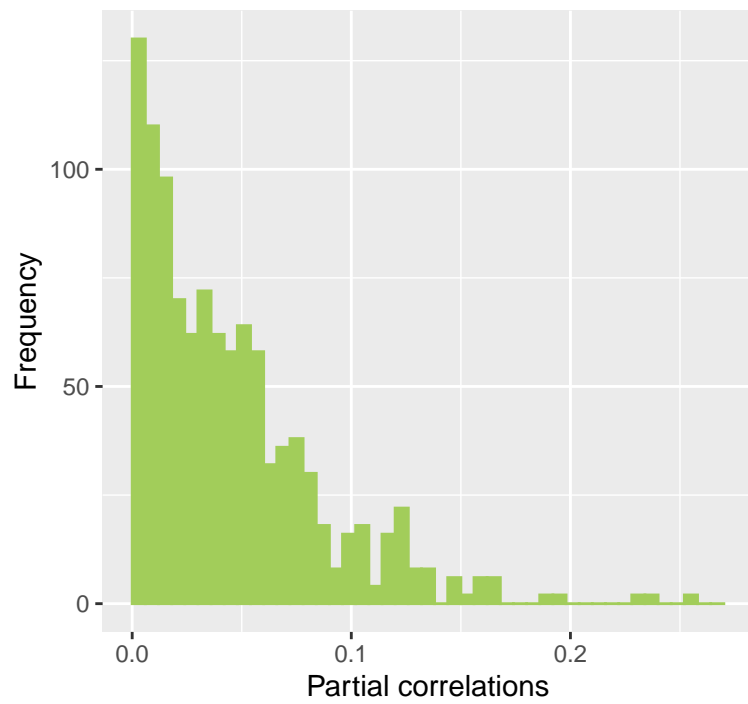

(d)

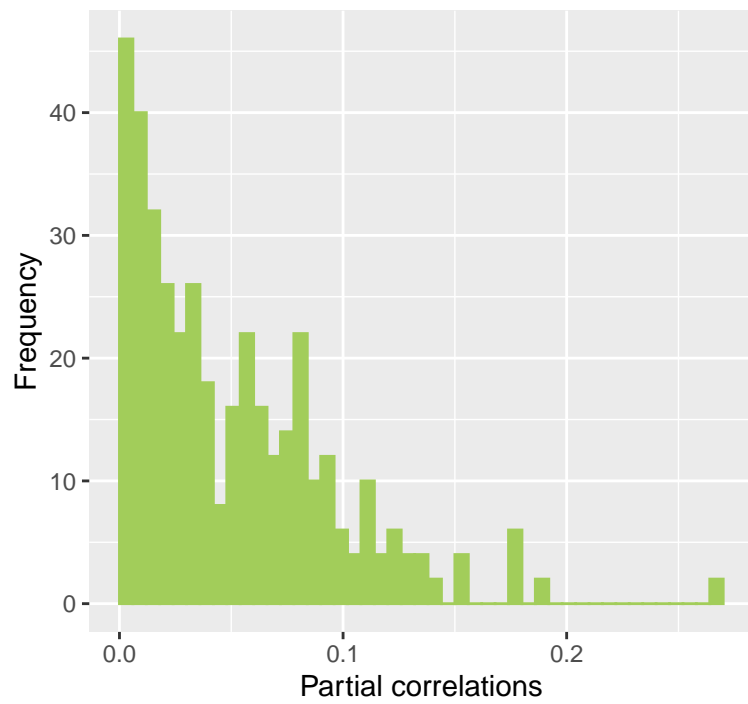

Supplement: Supplementary file 3 — Additional file 3. Comparison of histograms of the non-zero prior partial correlation weights for the simulated data and the real multiomic data. The histograms show how the distribution of the prior weights in our simulations resemble the distribution of the prior weights used in the multiomic applications. The histograms show the non-zero prior weights for the simulated data with partial correlations equal to (a) 0.02 and (b) 0.01, and the real genomic data from (c) the TCGA data set and (d) the Oslo 2 data set. [file 12859_2021_4413_MOESM3_ESM.pdf]

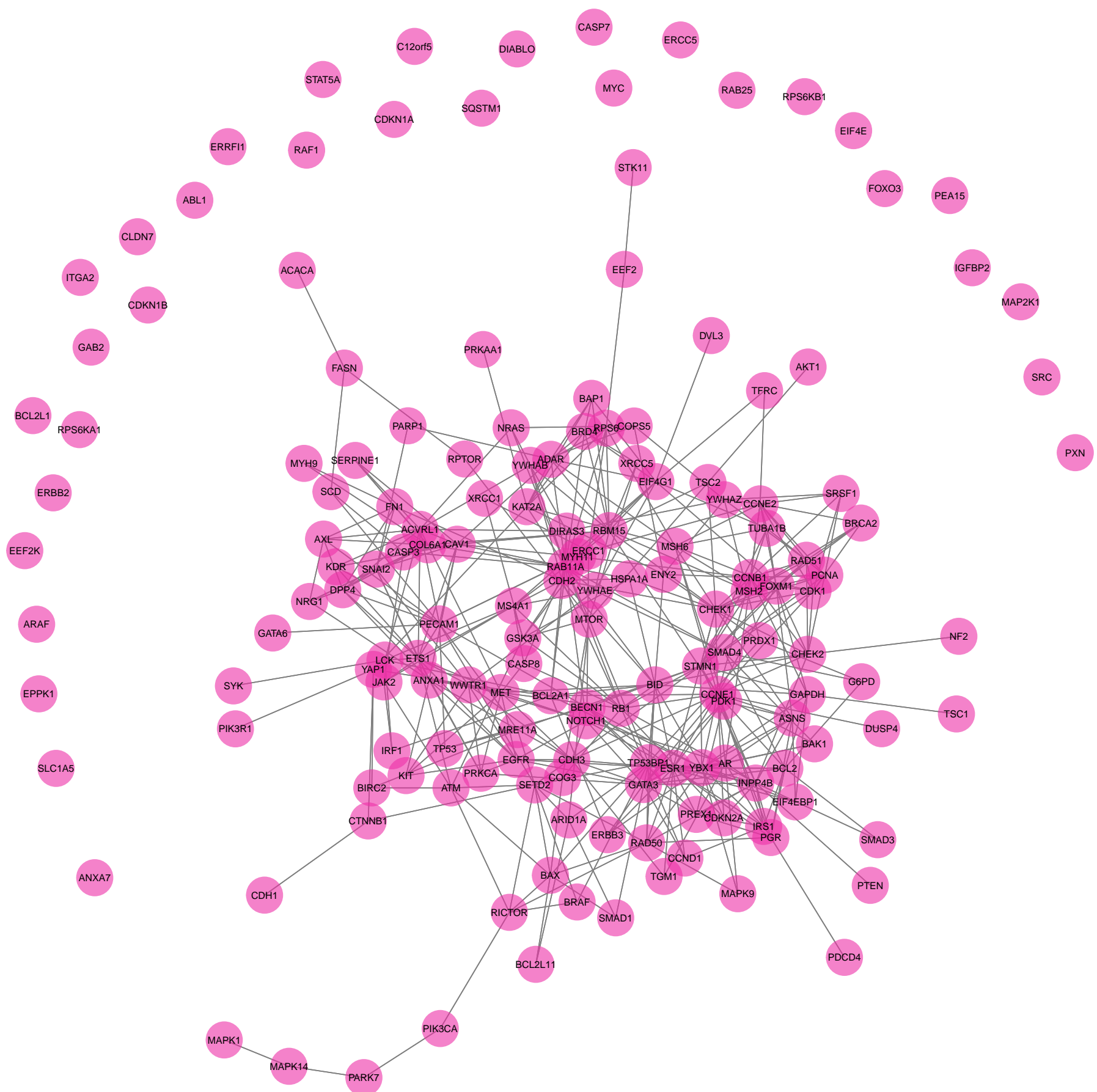

Supplement: Supplementary file 4 — Additional file 4. The tailored graphical lasso graph for the TCGA BRCA RPPA data. The graph found in the analysis of the TCGA BRCA data we did in this paper, using the RNA-seq data as prior information. [file 12859_2021_4413_MOESM4_ESM.pdf]

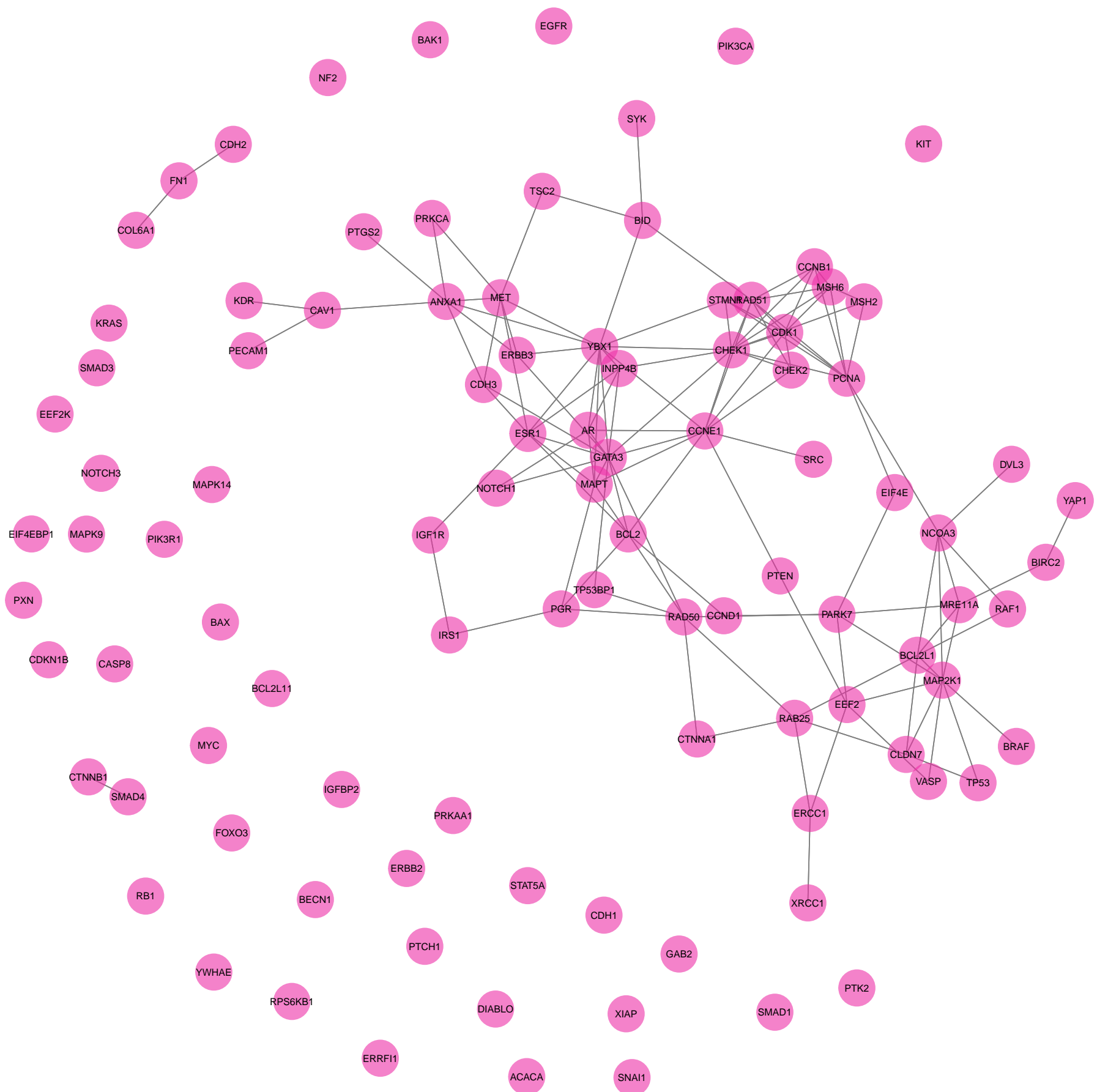

Supplement: Supplementary file 5 — Additional file 5. The tailored graphical lasso graph for the Oslo 2 RPPA data. The graph found in the analysis of the Oslo 2 data we did in this paper, using the mRNA data as prior information. [file 12859_2021_4413_MOESM5_ESM.pdf]
